# Polyploidy and Mutagenic Germplasm Innovation in Minor Legumes: Paradigm Shift and Challenges from Model Crops to Mung Bean

**DOI:** 10.3390/plants15071051

**Published:** 2026-03-29

**Authors:** Feixiang Guo, Chao Ma, Yuan Liu, Lixia Wang, Chunxia Li

**Affiliations:** 1 College of Agriculture, Henan University of Science and Technology, Luoyang 471023, China; gfx0405@163.com (F.G.); liuyuan07162001@163.com (Y.L.); lichx@haust.edu.cn (C.L.); 2 Institute of Crop Sciences, Chinese Academy of Agricultural Sciences, Beijing 100081, China

**Keywords:** mung bean, polyploid breeding, mutagenesis breeding, genetic improvement, paradigm shift, precision design breeding

## Abstract

Minor legume crops, including mung bean (*Vigna radiata*), cowpea, and adzuki bean, are crucial for global food security and sustainable agriculture, yet their genetic improvement has been hindered by narrow germplasm resources and lagging breeding technologies. This article systematically reviews the strategy of integrating polyploid breeding with mutagenic breeding as an innovative pathway to overcome the genetic bottlenecks of minor legumes. It focuses on insights gained from model plants and major legume crops like soybean and alfalfa regarding polyploid advantages and efficient mutagenesis techniques. Furthermore, it provides an in-depth analysis of the unique challenges and adaptation barriers encountered when transferring these paradigms to minor crops. Using mung bean as a representative case study, this review highlights specific challenges, including the creation of stable polyploid germplasm, the elucidation of complex regulatory mechanisms in polyploid genomes, and the technical bottlenecks in gene mapping and functional validation. The review also outlines future directions involving the integration of cutting-edge technologies—such as multi-omics, high-throughput phenomics, and gene editing—to establish a holistic research framework of “germplasm innovation-gene mapping-designer breeding”. This integrated approach aims to advance the breeding practices of minor legumes into a new era of precision design.

## 1. Introduction

### 1.1. Nutritional Value and Industrial Status of Minor Legume Crops

Legume orphan crops, encompassing species such as mung bean, cowpea, adzuki bean, faba bean, pea, and common bean, play indispensable roles in global agroecosystems and human nutrition [1]. They serve as vital sources of plant-based protein and dietary fiber, particularly in many developing regions. Their unique health-promoting value is attributed to the abundance of bioactive compounds, such as polyphenols and flavonoids [2]. Ecologically, their capacity for symbiotic nitrogen fixation with rhizobia establishes them as crucial components within sustainable agricultural systems. However, the genetic improvement and yield advancements of minor legumes progress much more slowly than those of major cereal staples (e.g., rice and maize) as well as major legume crops (e.g., soybean and pea). For context, the global production of major cereals exceeds billions of tons, and soybean production reaches approximately 390 million metric tons annually, whereas minor legumes remain a small fraction of these figures. Furthermore, the average yield of minor legumes often lags significantly behind that of soybean and major cereals, highlighting a severe “yield gap” [3,4]. Despite this gap, in the context of the growing global demand for nutritional health and climate resilience, the strategic importance of minor legumes is undergoing a paradigm shift—from a traditional “supplementary role” towards a “key pillar” in future food systems.

### 1.2. Limitations of Conventional Breeding: Narrow Genetic Base and Scarcity of Elite Germplasm

Despite strong industrial demand, the genetic improvement of legume orphan crops progresses much more slowly than that of major staples like rice and maize, as well as major legume crops such as soybean and pea. A primary bottleneck is their narrow genetic base, which results in a critical shortage of elite, breakthrough germplasm [5,6]. Prolonged domestication, intensive selection, and interspecific reproductive barriers have severely limited genetic diversity within cultivated gene pools, depleting the reservoir of superior alleles. Within this constrained genetic landscape, conventional hybridization-based breeding fails to achieve the synergistic gains in key agronomic traits-such as yield, quality, stress resilience, and mechanization-related traits like compact architecture, lodging resistance, and uniform maturity-that are necessary for a breeding revolution [7]. This situation, akin to “a skilled cook lacking ingredients”, directly impedes the development of transformative cultivars required for modern intensive, mechanized, and functionally diversified agriculture.

Nevertheless, it is important to emphasize that recent research on the *Vigna* genus is actively addressing these challenges by leveraging comparative genomics and functional analysis. A notable example is a 2025 study that successfully identified *VuCERK1*, a pattern recognition receptor (PRR) in cowpea. This gene, homologous to the well-characterized *AtCERK1* in *Arabidopsis* and *OsCERK1* in rice, was proven to play a dual role in both biotic defense and salt stress tolerance [8]. Such progress demonstrates that the advanced genetic knowledge from model crops is being effectively transferred to *Vigna* species, paving the way for targeted genetic improvement. It, therefore, represents a major barrier to industrial upgrading and to effectively addressing the challenges posed by climate change.

### 1.3. Unique Advantages of Polyploidy and Mutation Breeding in Expanding Genetic Variation

To break through the genetic constraints outlined above, creating novel and extensive genetic variation is the crucial first step. However, it must be emphasized that while these innovative techniques generate the raw genetic diversity, the fundamental steps of classical breeding—specifically, subsequent crossing and rigorous phenotypic selection—remain indispensable for effectively incorporating these novel alleles into elite cultivars. In this respect, polyploidy breeding and mutation breeding, as two classic and highly complementary strategies for generating this initial variation, present unique and significant advantages [9,10]. Polyploidy breeding, by duplicating the whole set of chromosomes, can induce large-scale, genome-wide changes, and is often associated with a set of “polyploidy effects” (e.g., organ gigantism, a significant increase in biomass, enrichment of secondary metabolites, and enhanced tolerance to abiotic stresses). It provides a unique operational framework at the chromosomal level, enabling the creation of germplasm with novel phenotypic profiles, such as forage types with enhanced biomass production and ecological types with superior stress resilience [11].

Mutation breeding, encompassing both chemical mutagenesis (e.g., EMS) and physical mutagenesis (e.g., gamma irradiation), on the other hand, can introduce random point mutations or structural variations into the genome with a relatively high frequency. This approach effectively mimics the process of natural variation, rapidly expanding allelic diversity. For example, recent studies in legume crops have successfully utilized physical mutagens like gamma irradiation to generate novel *Pisum sativum* (field pea) mutant populations exhibiting dual-purpose traits, such as enhanced tolerance to devastating fungal diseases (like *Ascochyta* blight) combined with improved yield-related characteristics [12]. Such robust strategies serve as a direct route for mining novel genes underlying key agronomic traits and for generating superior allelic variants that are rare or even absent in nature [13]. The combination of these two strategies offers comprehensive coverage of genetic perturbation, spanning from the macro-chromosomal (whole chromosome sets) to the molecular (single nucleotide) levels. Together, they constitute a powerful toolkit for systematically dissecting and reshaping crop phenotypes.

### 1.4. Using the Model Legume/Staple Crop Paradigm as a Reference: Proposing a “Paradigm Transfer” Pathway

“Paradigm transfer” in this context refers to the systematic translation of established translational genomics pipelines, data resources, and research frameworks from model systems to orphan crops. Unlike general model plants such as *Arabidopsis thaliana*, the specific evolutionary proximity of mung bean to model legumes, particularly soybean and *Medicago truncatula*, provides a rigorous scientific basis for this transfer. This strategy encompasses three core components:

First, the adaptation of precision breeding technologies. This involves calibrating established protocols from major legumes, including optimized methods for chromosome doubling and chemical mutagenesis (e.g., standardized optimization of colchicine concentration-time parameters in previous soybean research) [14]. By leveraging high-throughput phenotyping platforms originally developed for legume canopy architectures, we can develop standard operating procedures (SOPs) tailored for mung bean, thereby lowering the technical threshold for implementation.

Second, the exploitation of syntenic data resources. This entails leveraging existing genomic annotations, QTL mapping databases, and phenotype–gene association information from well-characterized legume species. Due to the high degree of genomic synteny between mung bean and soybean, researchers can utilize cross-species comparative genomics to rapidly identify orthologous genes in mung bean, significantly shortening the cycle for functional validation [15].

Third, the adaptive modification of methodologies. Techniques developed for model systems are tailored to the specific biological characteristics of mung bean. For example, TILLING platforms established for soybean can be adapted for screening mung bean mutant populations. Similarly, applying CRISPR/Cas9 for precise gene editing in polyploidized mung bean lines aims to overcome “technical incompatibility” by utilizing vector systems and promoters already validated in the Fabaceae family [16].

Importantly, the theoretical and technical frameworks for polyploidy and mutation breeding have matured into robust research paradigms. By integrating knowledge from general model plants (e.g., *Arabidopsis*) and, more critically, model legumes (e.g., soybean), these resources provide a foundational scaffold for the genetic improvement of orphan legumes like mung bean [17,18]. These paradigms encompass a complete research cycle, spanning from the efficient generation of mutants/polyploids and high-throughput phenotyping to multi-omics analyses and gene-editing validation. The central thesis of this review is that these advanced paradigms can serve as a foundational reference and starting point for systematically examining their application, effectiveness, and encountered bottlenecks during transfer to orphan legume crops, using mung bean as a representative case [19]. We aim to move beyond mere technological adoption. Instead, we will explore in depth how these paradigms can undergo “adaptive re-engineering” and “intelligent transfer”, guided by mung bean’s specific genomic architecture, reproductive biology, and industrial demands. The ultimate goal is to delineate an efficient and viable innovation pathway for the genetic improvement of orphan legume crops.

Furthermore, to provide better context, highlight existing research contributions, and clearly demonstrate the unique novelty of the present study, a comprehensive comparison between our work and previously published reviews on legume breeding and genomic tools is provided in Appendix A (Table A1). By bridging the translational gap from model crops to minor legumes, this review establishes a synergistic framework that modernizes classical mutagenesis and ploidy manipulation with advanced genomic paradigms.

### 1.5. Literature Search Strategy and Selection Criteria

To ensure methodological transparency and minimize selection bias, a comprehensive literature search was conducted using major academic databases, including Web of Science Core Collection, Scopus, and PubMed. The search strategy employed combinations of the following primary keywords: (“mung bean” or “*Vigna radiata*” or “minor legumes” or “orphan crops”) and (“polyploidy” or “mutagenesis” or “mutation breeding” or “genome editing” or “CRISPR”).

The inclusion criteria were restricted to peer-reviewed articles, high-quality reviews, and book chapters published primarily between 2010 and 2026, with seminal older papers included where historical context was necessary (e.g., early radiation mutagenesis). Studies focusing exclusively on major crops (like rice or maize) were only included if they provided a highly relevant technological paradigm with explicit potential for translation to recalcitrant legume systems.

## 2. Advances in Polyploidy Breeding of Legumes and Model Crops

### 2.1. The Role of Polyploidy in Crop Evolution and Domestication

Polyploidization represents a pivotal driver of plant evolution and species diversification, leaving profound imprints throughout the history of crop domestication. The origins of many modern staple crops, such as allohexaploid common wheat and oilseed rape, are closely associated with historical polyploidization events [20,21]. These events, involving the merger of genomes from distinct ancestral species, not only confer notable heterosis but also, based on genomic redundancy, facilitate subgenome specialization, interaction, and evolutionary innovation. This process has been instrumental in generating key agronomic traits, including enhanced grain yield, improved fiber quality, and altered oil composition. The successful domestication and widespread cultivation of these crops provide robust evolutionary-biological evidence for the immense potential of polyploidization in broadening genetic diversity, strengthening environmental adaptability, and creating novel phenotypes. This underpins the fundamental rationale and promising outlook for artificial polyploidy breeding [22].

While traditional approaches to artificial chromosome doubling have heavily relied on colchicine, modern methodologies have significantly diversified. Recent advancements, particularly in the Brassicaceae family, demonstrate that various other antimitotic agents can be equally or more effective. For instance, recent studies evaluating doubled haploid lines of swede have shown that alternative reagents such as ethalfluralin, trifluralin, and amiprophos-methyl yield higher percentages of chromosome doubling compared to traditional colchicine treatments [23]. The exploration of these diverse antimitotic agents provides a broader and more efficient toolkit for artificial polyploidization across various crop species.

### 2.2. Current Status of Polyploidy Research in Legumes

Within legume crops, polyploidy research presents a landscape where fundamental science and applied exploration proceed in parallel. Soybean, an ancient allopolyploid, has undergone a complex history of polyploidization followed by diploidization, serving as an excellent model for investigating the long-term evolution of polyploid genomes and the remodeling of their regulatory networks [24]. Research on artificially synthesized neo-allopolyploids in soybean aims to combine beneficial traits from wild relatives, but often faces significant challenges such as severe fertility reduction and genomic instability [25]. In contrast, the naturally occurring tetraploid alfalfa stands as a successful paradigm of legume polyploidy breeding. Its high yield, superior quality, and strong adaptability are attributed to precise mechanisms of advantageous complementarity and coordinated expression between its two subgenomes, a phenomenon termed “subgenome advantage” [26]. Deciphering the early adaptive barriers in artificial soybean polyploids and the mechanisms maintaining fitness in natural alfalfa polyploids constitute two cutting-edge research fronts for revealing the principles of trait formation in polyploids.

**Figure 1:**
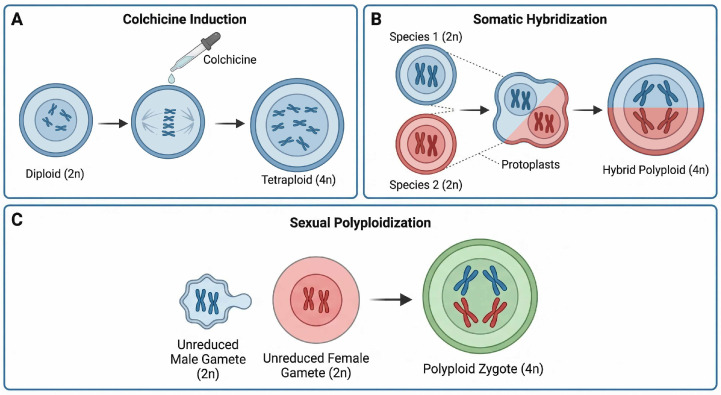
Schematic representation of primary technical pathways for artificial plant polyploidy induction. (**A**) Chemical induction: Chemical agents (e.g., colchicine, represented by green circles) inhibit microtubule polymerization (blocking spindle assembly), preventing chromosome segregation during mitosis and achieving chromosome doubling in a single cell. (**B**) Somatic hybridization: Protoplast fusion from different species (red and blue protoplasts) results in a hybrid cell with fused nuclei (and merged genomes). (**C**) Sexual polyploidization (via 2n gametes): Fusion of two unreduced gametes (both 2n) directly produces a tetraploid (4n) zygote. This figure was generated using the AI image synthesis model Nano Banana2 2K, based on prompts provided by the authors. The resulting images were verified for scientific accuracy by the authors.

The technical repertoire for artificially creating crop polyploids has become quite diverse, primarily encompassing chemical induction, somatic hybridization, and sexual polyploidization pathways (Figure 1) [27,28,29]. Colchicine induction remains the most classic and widely used method. It operates by inhibiting the formation of the mitotic spindle, preventing chromosome segregation after replication, thereby achieving chromosome doubling. While relatively straightforward, this method requires precise control of the treatment site, concentration, and duration (Figure 1A) [30]. Somatic hybridization technology, via protoplast fusion, enables the merging of genomes from distant species or even genera, overcoming pre-zygotic reproductive barriers and offering the possibility of creating novel cybrids. However, it is technically complex and often faces plant regeneration difficulties (Figure 1B) [28]. Additionally, utilizing unreduced gametes (2n gametes) for sexual hybridization is a pathway that more closely mimics the natural process of polyploid formation, though it is characterized by low frequency and challenges in precise artificial control (Figure 1C) [29]. Each of these methods possesses distinct advantages and limitations, and together they underpin the controlled creation of novel polyploid germplasm resources.

### 2.3. Phenotypic Effects and Breeding Roles of Polyploidy

**Figure 2:**
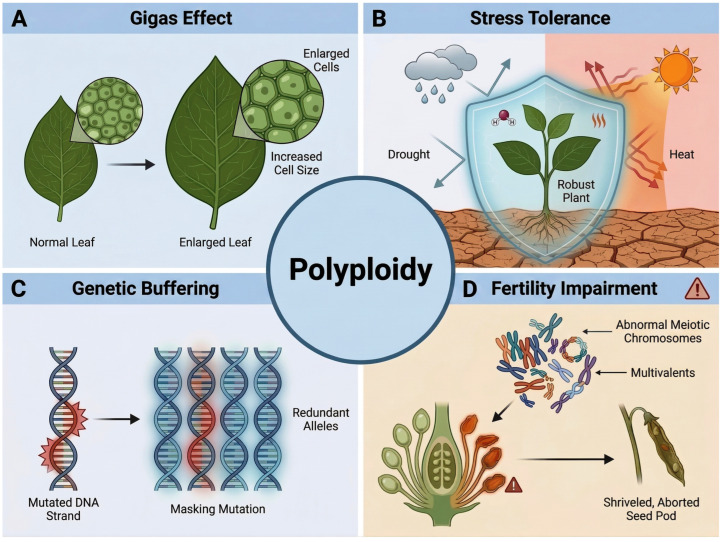
The multifaceted phenotypic effects and breeding value of plant polyploidy. (**A**) Gigas Effect: Polyploidization induces an increase in cell size, directly leading to enlarged vegetative organs (such as leaves) and a general accumulation of biomass, which contributes to higher agronomic yield. (**B**) Enhanced Stress Tolerance: Polyploids often develop thicker physical barriers and robust physiological defense systems (illustrated by the biological shield), conferring higher tolerance to abiotic stresses including drought and extreme heat. (**C**) Genetic Buffering: Genome duplication provides redundant alleles that can effectively mask the expression of deleterious recessive mutations (mutated DNA strand), allowing polyploids to endure higher doses of mutagens. (**D**) Fertility Impairment: As a significant evolutionary and breeding trade-off, polyploidy disrupts normal meiotic division. Abnormal homologous chromosome pairing leads to the formation of multivalents, which ultimately results in impaired floral organ development, shriveled/aborted seed pods, and low fertility. The figure was generated using the AI image synthesis model Nano Banana2 2K, based on prompts provided by the authors. The resulting images were verified for scientific accuracy by the authors.

Polyploidy induces a series of predictable phenotypic changes that serve as a novel genetic and morphological chassis for crop improvement (Figure 2). The most direct is the gigantism effect, characterized by increased cell volume leading to significantly enlarged organs such as leaves, flowers, fruits, and seeds, along with a general accumulation of biomass (Figure 2A) [31]. In breeding practices, this gigas effect directly contributes to target agronomic traits, such as higher forage yield or increased thousand-seed weight.

At the physiological and metabolic levels, polyploids often exhibit altered levels of secondary metabolites and enhanced tolerance to abiotic stresses (e.g., drought, salinity, extreme temperatures). The underlying mechanisms may involve the accumulation of osmolytes, activation of antioxidant systems, and reinforcement of stress-responsive signaling pathways resulting from larger cell volumes and thicker barriers (Figure 2B) [32].

Furthermore, at the genetic level, a crucial but often overlooked role of polyploidy is the “genetic buffering effect.” The duplication of the genome provides redundant alleles, which can mask the expression of deleterious recessive mutations. This buffering capacity allows polyploid plants to endure much higher doses of mutagens compared to diploids, making polyploidization an ideal pre-treatment for subsequent mutation breeding to generate ultra-high-density allelic variations (Figure 2C) [33].

However, the advantages of polyploidy are frequently accompanied by a significant trade-off-fertility impairment [34]. Chromosome doubling disrupts homologous chromosome pairing (forming multivalents) and segregation during meiosis, resulting in markedly reduced pollen viability, impaired embryo sac development, and ultimately leading to low seed set and poor seed development. This fertility barrier constitutes a primary constraint for the application of polyploidy in plant breeding (Figure 2D) [14].

### 2.4. The “Technology–Phenotype–Genome” Research Paradigm in Model Crops

In summary, systematic research on model species such as *Arabidopsis thalianaand* soybean has progressively established a relatively comprehensive, tripartite research paradigm for polyploidy: “Technology Development-Phenotypic Profiling-Genomic Deciphering” [17,18]. This paradigm begins with efficient and reliable ploidy induction and identification techniques. It then proceeds to a comprehensive phenomic evaluation of polyploid materials, encompassing morphology, physiology, yield, and stress resistance. Finally, it employs a suite of tools-including comparative genomics, transcriptomics, epigenomics, and proteomics-to elucidate the causal links between macro-level phenotypes and underlying molecular events. These events occur across multiple layers, such as genomic structural variation, gene dosage effects, allelic interactions, and epigenetic reprogramming. This systematic framework provides a solid foundation for elevating polyploid breeding from an empirical practice into a predictable and designable science. It also offers a clear roadmap for transferring this advanced paradigm to other crops.

## 3. Advances in Mutation Breeding in Legume and Model Crops

### 3.1. Evolution of Mutagenesis Techniques

**Figure 3:**
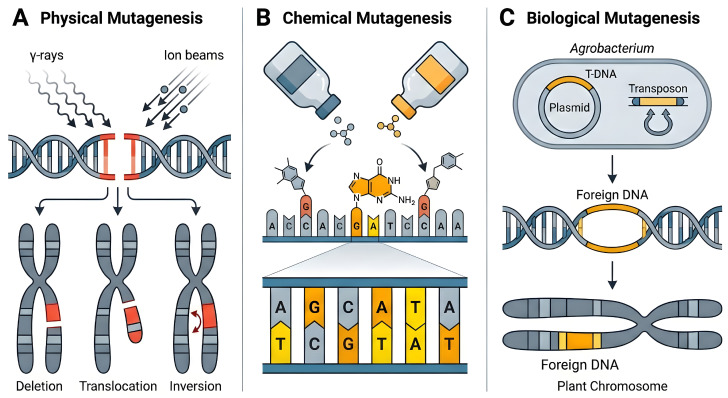
Schematic representation of primary technical approaches and mechanisms in plant mutation breeding. (**A**) Physical Mutagenesis: Physical agents such as gamma rays or ion beams induce DNA double-strand breaks. Subsequent DNA repair processes can generate structural variations, including chromosomal deletions, translocations, or inversions. (**B**) Chemical Mutagenesis: Chemical mutagens (e.g., EMS) specifically modify DNA bases, leading to base mispairing during replication (e.g., G/C to A/T substitution) and thus generating point mutations. (**C**) Biological Mutagenesis: Biological elements, such as Agrobacterium T-DNA or transposons, are used to integrate foreign DNA fragments into the plant genome, causing gene disruption or alteration and resulting in insertion mutations. This figure was generated using the AI image synthesis model Nano Banana2 2K, based on prompts provided by the authors. The resulting images were verified for scientific accuracy by the authors.

Mutation breeding, which artificially induces genomic mutations through physical, chemical, or biological means, serves as a powerful tool for creating genetic variation and driving germplasm innovation (Figure 3). The development of mutagenesis techniques follows a distinct trajectory, with each category offering unique advantages. Physical mutagens, such as gamma rays and ion beams, primarily induce DNA double-strand breaks, thereby generating large-scale structural variations like chromosomal deletions, translocations, and inversions (Figure 3A) [35]. Chemical mutagens, exemplified by alkylating agents like ethyl methanesulfonate (EMS) and sodium azide, predominantly cause base substitutions (e.g., G/C to A/T). Their suitable mutation spectra and subsequent amenability to gene cloning make them the preferred choice for constructing large-scale, saturated mutant populations (Figure 3B) [36]. Biological mutagenesis primarily utilizes Agrobacterium tumefaciens-mediated T-DNA insertion or transposon tagging to generate stable insertion mutations in model plants (e.g., *Arabidopsis*, rice), serving as a robust tool for functional genomics studies (Figure 3C) [37]. The development and integration of these techniques enable researchers to selectively create genetic variations of different scales and types according to their needs.

In legume model plants, the systematic construction of mutant populations has provided a core resource for gene function elucidation. In Medicago truncatula, a large-scale, screenable mutant population has been established through EMS chemical mutagenesis coupled with high-throughput TILLING technology [38]. Researchers can rapidly retrieve allelic mutants of target genes from this population via PCR-based screening, enabling efficient reverse genetics studies. Lotus japonicus leverages its highly efficient genetic transformation system to construct large-scale T-DNA insertion mutant libraries, which have played a pivotal role in deciphering the molecular mechanisms of legume-specific traits, such as nodulation, nitrogen fixation, and leaf movement [39]. The key genes identified in these model systems not only deepen our understanding of fundamental legume biology but also provide valuable candidate gene targets for molecular design breeding in crops like soybean and mung bean.

### 3.2. Successful Cases of Mutation Breeding in Staple Food Crops

Successful applications of mutation breeding in staple crops such as rice and barley (Table 1) fully demonstrate its substantial practical value in directly developing elite cultivars. The discovery of the semi-dwarf *sd1* gene, a cornerstone of the “Green Revolution”, originated from the screening of natural and radiation-induced mutants [40]. Through mutagenesis using agents like gamma rays or EMS, combined with meticulous phenotypic selection, a series of new rice and barley varieties harboring valuable agronomic traits have been successfully developed. These traits include semi-dwarfism, early maturity, glutinous endosperm, low phytic acid content, and improved storage tolerance, with many varieties directly deployed in production [41,42]. These cases underscore that mutagenesis serves not only as a tool for fundamental research but also as a highly efficient breeding strategy capable of directly driving cultivar improvement.

### 3.3. Evolution of High-Throughput Mutant Screening Strategies

Advancements in technology have driven the evolution of mutant screening strategies from broad-based to precise paradigms. Early efforts primarily relied on “Phenotype-Driven Screening”which involved visually inspecting or performing simple measurements on large populations to identify visible morphological, physiological, or resistance-related mutations-a classical forward genetics approach [56]. The advent of TILLING technology marked the onset of the “Gene-Driven Screening” era. This technique enables researchers to screen for mutants in a specific gene sequence without prior knowledge of the phenotype, facilitating reverse genetics operations from gene to phenotype [57]. The maturation of CRISPR/Cas9-mediated gene editing and the application of its library screening strategies have further propelled mutagenesis from a “random” process to a new era of “Precision-Targeted Creation”.This development allows for the systematic functional knockout and phenotypic analysis of specific gene families or signaling pathways [58].

### 3.4. Paradigm Shift in Mutation Breeding: From “Random Screening” to “Precision Creation”

Reflecting on its developmental trajectory, mutation breeding has evolved from an initial model reliant on chance and large-scale phenotypic screening—characterized as “random mutation, phenotypic selection”—to the contemporary paradigm of “design-oriented, precision creation”, deeply integrated with high-throughput sequencing, bioinformatics, high-throughput phenotyping, and precise gene editing [56]. Traditional mutagenesis techniques retain irreplaceable value for expanding the genetic base, owing to their advantages of lower cost, genotype independence, and ability to generate diverse variations. This is particularly relevant for crops recalcitrant to genetic transformation. In parallel, modern molecular technologies provide powerful tools for the rapid identification of mutant genes, functional validation, and even the targeted creation of desired allelic variants. The organic integration and iterative advancement of these traditional and modern technologies together constitute the comprehensive toolbox and strategic direction for current research in crop mutation breeding.

## 4. Complementary and Synergistic Integration of Polyploidy and Mutation Breeding: Encompassing Genetic Perturbations from Macroscopic Chromosomes to Microscopic Bases

Polyploidy breeding and mutation breeding serve as classical strategies for broadening genetic variation. Research in model crops has revealed their significant complementarity and synergistic potential, fundamentally rooted in their differences and mutual reinforcement across three dimensions: the scale of genetic perturbation, the underlying variation mechanisms, and the resulting phenotypic effects.

### 4.1. Complementarity in the Scale of Genetic Perturbation

Polyploidy breeding induces macro-scale genomic upheaval through whole-genome duplication, often accompanied by “polyploidy advantages” such as organ gigantism, increased biomass, and enhanced abiotic stress tolerance. This approach provides a chromosomal-level platform for creating novel phenotypes (e.g., high-biomass forage types, stress-resistant ecotypes) [31]. In contrast, mutation breeding—particularly chemical mutagenesis using EMS—primarily generates molecular-level, random mutations at the single-base level. It efficiently mimics natural variation, rapidly expands allelic diversity, and serves as a direct pathway for mining novel genes controlling key agronomic traits or creating rare, superior allelic variants not commonly found in nature [36]. The combination of these two strategies achieves comprehensive coverage of genetic perturbations, spanning from the cytogenetic scale (whole chromosomes) to the molecular level (single nucleotides). This integrated approach provides a powerful toolkit for systematically deconstructing and reshaping crop phenotypes.

### 4.2. Synergy in Variation Mechanisms and Phenotypic Effects

The genomic redundancy and subgenome interactions resulting from polyploidization can alter gene regulatory networks, thereby enhancing or attenuating specific metabolic pathways. This creates a novel genetic background for point mutations generated by mutagenesis. For instance, within a polyploid context, certain recessive beneficial mutations induced by mutagenesis may become more readily observable due to gene dosage effects, or the deleterious effects of harmful mutations could be masked through subgenome complementation. Conversely, mutagenesis techniques can be employed to specifically create particular allelic variants that are absent in polyploid materials, or to edit key genes to rectify common fertility impairments in polyploids. This synergy enables the “strengthening of advantages and repairing of defects” for collaborative crop improvement [34].

### 4.3. Deep Integration in Technological Paradigms

The well-established “Technology–Phenotype–Genome” research paradigm in model crops provides a methodological foundation for the synergy between polyploidy and mutation breeding. High-throughput phenomics enables the simultaneous characterization of both the gigantism effects in polyploids and the subtle phenotypic variations in mutants [59]. Multi-omics technologies can uncover the interactions between epigenetic reprogramming triggered by chromosome doubling and cis-regulatory alterations caused by point mutations. Furthermore, gene-editing technologies such as CRISPR/Cas9 allow for the precise introduction of mutagenic allelic variants directly into polyploid genomes, facilitating a novel design-breeding model characterized by a “polyploid chassis + precision modification” strategy [60]. This deep integration drives the evolution of breeding paradigms from “random screening” towards “precision creation”, offering a systematic solution for overcoming the genetic bottlenecks in orphan legume crops.

## 5. Polyploid Germplasm Development and Characterization in Mung Bean

### 5.1. Genomic Features and Karyotype of Mung Bean

Mung bean (*Vigna radiata*), an important orphan legume crop, possesses several attributes that make it a suitable model for genetic studies. It has a relatively small genome (~579 Mb), a clear chromosome base number (2n = 2x = 22), and multiple high-quality, chromosome-level reference genome sequences are available [61]. This well-defined diploid background provides a clear starting point and ploidy controls for artificially induced chromosome doubling, establishing mung bean as an excellent system to investigate genomic, phenotypic, and adaptive changes during polyploidization in legume crops. It therefore exhibits significant potential for polyploidy research [62].

### 5.2. Artificial Polyploid Induction Techniques

The induction of artificial polyploids in mung bean primarily relies on classical methods, with colchicine treatment being the most common. Typical treatment targets include shoot apical meristems or germinating seeds. Chromosome doubling is achieved by soaking or applying a solution of colchicine at specific concentrations (typically 0.05–0.2%) to disrupt the mitotic process [63]. Treated plants often exhibit chimerism, containing both diploid and tetraploid cells. Consequently, rapid and accurate ploidy identification using flow cytometry is critical [64]. Subsequently, stable, genetically uniform autotetraploid lines must be isolated and purified from these chimeras through multiple generations of selfing or asexual propagation methods (e.g., stem cuttings). While sexual polyploidization pathways, such as utilizing unreduced gametes, are theoretically feasible, their practical application in mung bean remains exploratory.

### 5.3. Phenotypic Characterization of Polyploids

A series of phenotypic changes commonly observed in polyploid plants can be detected in artificially induced autotetraploid mung bean. In vegetative organs, leaves typically exhibit trends toward increased thickness and expanded surface area. Concomitantly, stomatal density often decreases, while the volume of individual guard cells tends to increase. These morphological characteristics are frequently utilized as preliminary indicators for ploidy identification [65,66]. Reproductive organs are also affected, manifesting as increased flower size and pollen grain volume. Regarding yield-related traits, a notable phenomenon is seed “gigantism”, where the hundred-seed weight often increases. However, this may be accompanied by a reduction in pods per plant or seeds per pod [65]. These phenotypic variations provide initial clues for identifying mung bean polyploid materials, although their stability and expressivity can vary depending on genotype and treatment conditions.

### 5.4. Physiological and Stress-Tolerance Responses

The physiological and metabolic performance of mung bean polyploids is potentially more complex. Although increased chlorophyll content has been observed in some polyploids, the net photosynthetic rate is regulated by multiple factors including stomatal conductance, mesophyll conductance, and biochemical processes. Consequently, the biomass advantage in polyploids may stem more from the enlargement of organs (e.g., leaves) rather than a fundamental enhancement of photosynthetic efficiency per unit area [65].

Regarding abiotic stress tolerance, drawing on research from other crop polyploids, mung bean autotetraploids may enhance their tolerance by activating similar physiological adaptation mechanisms. For instance, studies across various plant polyploids have demonstrated that stress can induce the accumulation of osmolytes (e.g., proline), upregulate antioxidant enzyme system activity, and trigger adaptive changes in leaf structure (e.g., epidermal wax or cuticle) [67,68]. Preliminary observations in mung bean suggest that tetraploid materials might exhibit superior physiological responses to drought or salt stress compared to their diploid progenitors. However, the specific molecular mechanisms, regulatory networks underlying this qualitative observation, and the stability of these responses across varying stress intensities and developmental stages, require systematic validation through well-designed controlled experiments.

### 5.5. Fertility and Seed-Setting Impairment

Consistent with the widespread challenge faced by many artificially synthesized crop polyploids, reproductive impairment constitutes a core constraint for the agronomic application of mung bean polyploids. Chromosome doubling typically disrupts the normal progression of meiosis. Based on studies in model crops like Arabidopsis and rice, as well as other legume polyploids, abnormal homologous chromosome pairing (e.g., multivalent formation) and irregular segregation are frequent occurrences. This directly leads to the production of a high proportion of infertile gametes, manifesting as significantly reduced pollen viability [69]. Concurrently, the development of the female gametophyte (embryo sac) can also be impaired [70]. The combined consequence is low success rates of pollination and fertilization, a sharp decline in seed set, and frequent occurrences of seed abortion or underdevelopment in the seeds that do form [71].

Therefore, overcoming the severe fertility barrier caused by meiotic instability is the primary scientific problem that must be addressed to translate the potential advantages of mung bean polyploids in vegetative growth or stress tolerance into stable, heritable agronomic yield gains. Research targeting this issue can draw upon strategies explored in other crops, such as modulating chromosome behavior, employing sexual polyploidization (utilizing 2n gametes), or using gene editing to precisely modify key fertility-related genes.

## 6. Development of Mutagenized Germplasm and Construction of Mutant Libraries in Mung Bean

### 6.1. Optimization of Mutagenesis Treatment Conditions

The development of mutagenized mung bean germplasm typically begins with a preliminary exploration of mutagenesis conditions. Due to the scarcity of standardized empirical protocols across diverse mung bean cultivars, researchers frequently extrapolate baseline parameters from taxonomically related legumes. For the chemical mutagen EMS, researchers often test concentration gradients (e.g., 0.3% to 1.0%) combined with duration gradients (e.g., 8 to 16 h). In related legumes such as cowpea and soybean, pre-soaking seeds in water or buffer is a demonstrated critical step to increase seed coat permeability [72,73], a principle conceptually applied to mung bean. The goal is to find a balance between inducing sufficient mutation frequency and maintaining an acceptable survival rate (LD50) in the M1 generation. Studies in chickpea have demonstrated that sensitivity to mutagens varies significantly across genotypes, necessitating tailored empirical optimization even within the same species [74]. For physical mutagenesis (e.g., gamma rays), optimal dosage ranges established for common bean often serve as extrapolated guidelines for building initial mung bean mutant populations, though species-specific empirical validation remains essential [75].

### 6.2. Strategies for Constructing M1-M3 Populations

**Figure 4:**
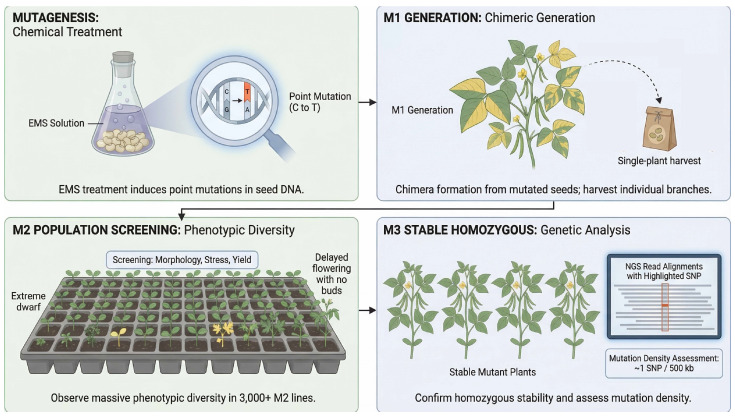
Generational flowchart for constructing an EMS—induced mutant library in mung bean (M1–M3 generations). Mutagenesis Treatment: Mung bean seeds are soaked in an EMS solution to induce genomic point mutations (predominantly C→T transitions). M1 Generation (Chimeric Plants): M1 plants develop as genetic chimeras (due to somatic mutations during seed development); seeds are harvested from individual branches/plants to establish M2 families (enabling mutant allele tracking from chimeric tissues). M2 Population Screening: M2 families segregate for recessive mutations. Plants with visible phenotypic alterations (e.g., chlorophyll deficiency, dwarfism, altered leaf shape, delayed flowering) are selected for further analysis (mass phenotypic diversity is observed across 3000 + M2 lines). M3 Stable Homozygous: M3 plants are grown to confirm trait homozygosity and genetic stability. Resequencing assesses mutation density (e.g., ~1 SNP/500 kb, consistent with theoretical saturation targets for mung bean, analogous to EMS—induced mutation densities in legumes like soybean). This figure was generated using the AI image synthesis model Nano Banana2 2K, based on prompts provided by the authors. The resulting images were verified for scientific accuracy by the authors.

The construction of a mung bean mutant library generally follows the widely adopted M1-M3 generation strategy (Figure 4). While large-scale, empirically validated mutant libraries are still limited in mung bean compared to major crops, drawing parallels from high-density TILLING populations in legumes like soybean and pea provides a conceptual framework. A library with practical screening potential should consist of several thousand M2 families to ensure adequate coverage [16,76]. M1 plants are treated as genetic chimeras, and seeds are harvested on a single-plant basis. In soybean, empirically verified mutation densities achieved through EMS typically range from 1 mutation per 140 kb to 1 per 550 kb [16]. For mung bean, given its smaller genome (~500 Mb), achieving a similar saturation level would theoretically require an M2 population of at least 3000 to 5000 independent lines. It must be noted, however, that these target population sizes remain conceptual projections; extensive empirical sequencing of mung bean mutant populations is still urgently needed to confirm actual mutation densities. 

### 6.3. Types of Phenotypic Variation

**Figure 5:**
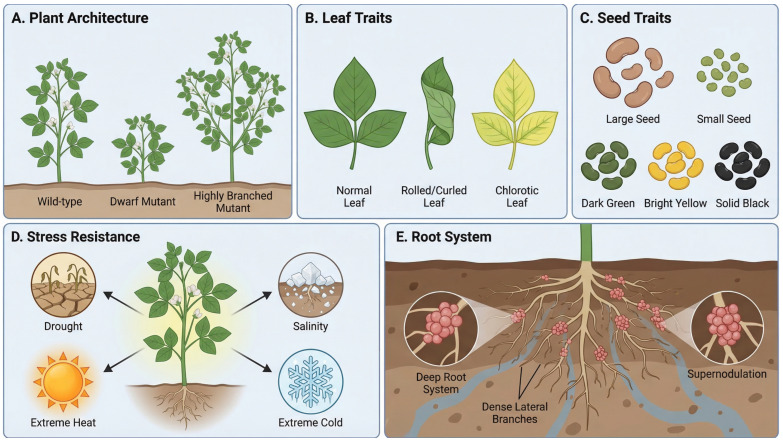
Extensive phenotypic variations generated through mutation breeding for crop improvement. (**A**) Plant Architecture: Mutagenesis induces significant alterations in plant structure, including dwarfism, tall stature, and modified branching habits compared to the wild type, which directly influence planting density and yield. (**B**) Leaf Traits: Variations in leaf morphology and pigmentation, illustrating mutants with rolled or narrow leaves, as well as altered leaf color such as chlorosis (yellowing) and variegation. (**C**) Seed Traits: Diversity in seed characteristics, highlighting variations in seed size (large or small seeds) and striking differences in seed coat color (e.g., dark green, yellow, solid black), which are often associated with altered seed quality (protein or starch composition). (**D**) Stress Resistance: Screening for robust mutants capable of withstanding severe abiotic stress conditions. The central healthy plant demonstrates enhanced tolerance to drought (cracked soil), extreme temperatures (heat and cold/freezing), and soil salinity. (**E**) Root System Architecture: Crucial underground trait variations showcasing a robust, deeper root system with high lateral root density for efficient water/nutrient uptake, alongside a “supernodulation” mutant featuring massive clustered root nodules essential for enhanced symbiotic nitrogen fixation in legumes. The figure was generated using the AI image synthesis model Nano Banana2 2K, based on prompts provided by the authors. The resulting images were verified for scientific accuracy by the authors.

Reported mutagenesis studies have revealed extensive phenotypic variations encompassing a wide range of agronomic traits (Figure 5). In established mung bean mutant populations, empirical observations frequently include alterations in plant architecture, such as dwarfism and changes in branching habit (Figure 5A) [77]; mutations affecting leaf morphology and color (Figure 5B) [78,79]; and distinct variations in seed coat color (e.g., dark green, yellow, black) (Figure 5C) [80]. Furthermore, screening for biotic and abiotic stress resistance has identified materials showing demonstrated potential against environmental adversities, including drought and salinity (Figure 5D) [81,82].

Critically, phenotypic variation is not limited to above-ground organs. While empirical screening for root system architecture (RSA) in mung bean mutants is still emerging, a robust root system is fundamental for nutrient uptake and stress tolerance [83]. Extrapolating from well-documented cases in soybean, altered root traits directly influence salt tolerance [84], and screening for “supernodulation” mutants represents a highly promising conceptual target for enhancing symbiotic nitrogen fixation in mung bean under suboptimal soil conditions (Figure 5E) [85].

### 6.4. Application Potential of High-Throughput Phenotyping Technologies

Applying High-Throughput Phenotyping (HTP) technologies represents a critical frontier for improving screening efficiency. Currently, the empirical application of HTP directly in mung bean mutant screening remains limited. However, recent studies in related legumes demonstrate the proven power of these tools, offering validated technological paradigms that could theoretically be transferred to mung bean. For instance, color and texture features derived from early-season canopy images have been successfully utilized to predict end-season yield in soybean [86]. Multispectral sensors on unmanned aerial systems have enabled the non-destructive estimation of biomass in field pea [87]. Furthermore, advanced three-dimensional reconstruction has been pioneered in related *Vigna* species (e.g., *Vigna subterranea*) to accurately quantify light interception [88]. Adapting these validated HTP pipelines represents a conceptual but highly feasible strategy to accelerate the characterization of mung bean germplasm under diverse environments.

## 7. Genetic Mapping of Key Traits in Mung Bean Polyploids and Mutants

The genetic dissection of key traits in mung bean polyploids and mutants is a crucial step linking germplasm development with breeding application. This process typically begins with constructing genetic populations, such as segregating populations for polyploids or near-isogenic lines for mutants. The development of molecular marker systems, evolving from SSRs to high-density SNPs, has provided the foundation for mapping efforts [89,90]. Based on this, studies have attempted preliminary QTL mapping for traits like leaf shape, flowering time, and plant height in mung bean, revealing polygenic control characteristics [91,92]. However, compared to model crops like rice and soybean, there are very few cases in mung bean where genes have been successfully map-based cloned and functionally validated. Current research often relies on bioinformatics strategies like homology-based comparisons for candidate gene prediction.

Despite these limitations, recent advances have successfully identified and functionally characterized several key genes governing vital agronomic and adaptive traits in mung bean. For example, regarding domestication traits, the *PDH1* gene has been recently deciphered as a crucial factor controlling pod dehiscence, providing a vital target for mechanized harvesting [93]. In terms of abiotic stress, overexpression of the mung bean E2 ubiquitin-conjugating enzyme gene, *VrUBC1*, has been proven to significantly enhance osmotic stress tolerance [94]. Furthermore, integrated multi-omics approaches have unraveled the molecular mechanisms underlying seed coat color variation by identifying key genes regulating anthocyanin accumulation [95]. Integrating these specifically mapped functional genes into the polyploid and mutagenic evaluation framework will provide exact molecular targets for future CRISPR/Cas9 editing and trait stacking.

For polyploid mung bean, genetic mapping is further complicated by issues such as genome stability and subgenome differentiation [96]. Drawing insights from studies on other polyploid crops, newly synthesized mung bean autotetraploids may also experience genomic rearrangements and homoeolog expression bias (subgenome dominance), which are important aspects for understanding their unique phenotypes [97]. Overall, this field faces two major challenges. The first is insufficient mapping resolution, limited by population size, recombination events, and the lack of high-quality pangenome references, resulting in excessively broad QTL intervals. The second is a weak functional validation system, where inefficient genetic transformation and immature gene editing technologies make it difficult to definitively confirm candidate gene function in mung bean itself. This current state of affairs, characterized by “imprecise mapping and inaccessible validation”, severely hinders the process of extracting applicable breeding knowledge from the rich phenotypic variations. Future breakthroughs are urgently needed in key areas such as expanding population sizes, constructing pangenomes, and overcoming transformation bottlenecks.

## 8. Paradigm Transfer from Model Crops to Mung Bean

### 8.1. Technology Transfer: Standardization and Scaling

**Figure 6:**
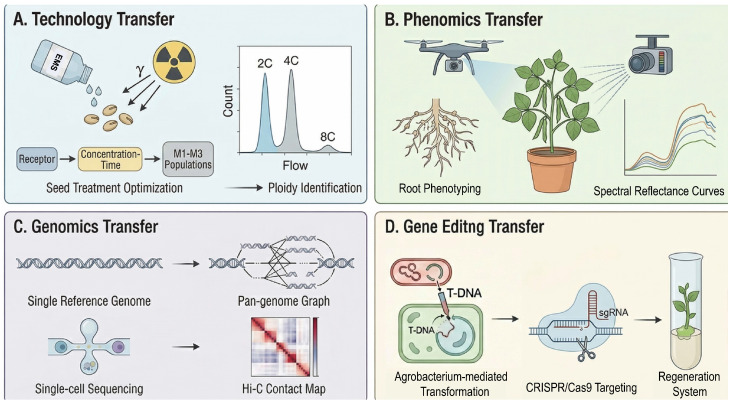
Paradigm transfer of advanced research systems to mung bean. (**A**) Technology transfer: Establishing standardized protocols for physical/chemical mutagenesis and flow cytometry-based ploidy identification to enable scalable germplasm development. (**B**) Phenomics transfer: Utilizing high-throughput imaging platforms (e.g., drones, multispectral cameras) to dissect complex traits in root and canopy architectures. (**C**) Genomics transfer: Evolving from reliance on a single reference genome to constructing pangenome maps, and integrating cutting-edge technologies such as single-cell sequencing and Hi-C to reveal genetic diversity and structure. (**D**) Gene editing transfer: Aiming to overcome the bottleneck of genetic transformation by establishing efficient Agrobacterium-mediated CRISPR/Cas9 precision editing and plant regeneration systems. This figure was generated using the AI image synthesis model Nano Banana2 2K, based on prompts provided by the authors. The resulting images were verified for scientific accuracy by the authors.

Transferring mature protocols for polyploid induction and mutagenesis from model crops to mung bean requires, as a primary task, achieving standardization and scaling (Figure 6A). Currently, empirical evidence regarding the optimal parameters for mung bean remains fragmented. This involves the systematic optimization of key parameters, such as identifying the optimal explant for colchicine treatment and establishing a standard operating procedure for rapid ploidy identification [16,98]. For mutagenesis, rather than relying solely on extrapolations from model crops, it is necessary to empirically clarify the differential sensitivity of major mung bean genotypes to agents like EMS. This transfer is not a mechanical replication but a parameter optimization based on the specific biological characteristics of mung bean. For instance, recent successful case studies in soybean demonstrated that optimizing the EMS concentration specific to seed size significantly increased mutation density [99]. While this provides a robust conceptual framework, generating equivalent empirical data for mung bean is the critical next step to establish a standardized, predictable breeding workflow.

### 8.2. Phenomics Transfer: From “Naked Eye” to “Intelligent Eye”

High-throughput phenomics technologies have emerged as the “intelligent eye” in model crop research (Figure 6B) [86]. Although the application of these technologies in mung bean remains in its nascent stage, their theoretical transfer entails the widespread adoption of automated imaging systems and root phenotyping platforms. This approach not only significantly enhances the efficiency and accuracy of screening for complex traits but also generates vast phenotypic datasets that are challenging to capture through traditional methods. Such data provide unprecedented support for establishing high-dimensional phenotype-genotype associations [100]. For instance, modern high-throughput platforms integrating 3D and RGB imaging (e.g., LeasyScan) have been successfully piloted in related legume crops such as chickpea and cowpea to monitor canopy dynamics [101]. These successful case studies validate the feasibility of applying “intelligent eye” technologies across the broader *Vigna* genus. However, extensive empirical validation under field conditions is still required to translate these conceptual models into practical screening tools specifically for mung bean polyploids and mutants.

### 8.3. Genomics Transfer: From “Reference Genome” to “Pangenome”

Although a reference genome is available for mung bean, a single genomic sequence cannot capture the rich intraspecific genetic diversity of this species. The core of the next stage in genomics transfer lies in the construction of a mung bean pangenome (Figure 6C). By performing deep sequencing of multiple representative accessions, the full spectrum of gene presence-absence variation can be comprehensively revealed. This will substantially improve the precision of gene mapping, particularly for genes absent from the reference genome [102]. Additionally, the application of single-cell sequencing technology to mung bean holds promise for resolving the transcriptomic dynamics of specific cell types during polyploid reproductive development [103]. Meanwhile, three-dimensional genomics techniques could help elucidate the effects of chromosome doubling on higher-order chromatin structures and the long-range gene interactions they regulate [104]. The implementation of these cutting-edge genomic technologies will elevate the genetic dissection of mung bean to a new level of resolution. The soybean pangenome project serves as a compelling example, having captured thousands of structural variations absent from the reference genome, many of which are associated with key domestication traits such as seed coat color and flowering time. This demonstrates that expanding from a single reference genome to a pangenome is not only theoretically significant but also essential for comprehensively capturing the genetic diversity of leguminous species [105]. However, it must be emphasized that the application of these advanced technologies in mung bean currently remains a conceptual goal; extensive methodological optimization is required before they can yield empirical insights.

### 8.4. Gene Editing Transfer: From “Hurdles” to “Breakthroughs”

The CRISPR/Cas9 gene editing technology represents the ultimate paradigm for precision creation, yet its successful application in mung bean currently faces significant obstacles [106]. The core empirical bottleneck is the extremely low, genotype-dependent genetic transformation efficiency caused by traditional *Agrobacterium*-mediated methods. Future research must focus on screening highly competent genotypes and optimizing tissue culture regeneration systems to overcome this limitation. Once efficient genetic transformation is achieved, this technology can be utilized to precisely modify the genome of polyploid mung bean. For example, targeted editing strategies for key genes associated with fertility defects—although currently still at the conceptual stage—hold the potential to “cure” the inherent defects of polyploids and enable the directed stacking of advantageous traits [34].

To overcome the tissue culture bottleneck, emerging “regeneration-bypass” delivery technologies have emerged. However, it is crucial to clearly distinguish between the empirical achievements of these technologies in other plant systems and their hypothetical applications in leguminous crops. For instance, advanced nanomaterial-mediated delivery has achieved notable breakthroughs: recent studies have successfully utilized carbon nanotubes to achieve non-integrating DNA delivery in intact plants [107], designed exosome/liposome-like nanoparticles as novel carriers [108], and developed mesoporous silica nanoparticles as biocompatible platforms to effectively protect CRISPR cargos from cellular nucleases, enabling delivery into recalcitrant plant genotypes [109]. At present, however, the application of such advanced nanocarriers in mung bean lacks empirical evidence and remains strictly at the stage of theoretical deduction. The unique cell wall composition and dense epidermal layers characteristic of legumes constitute physical barriers that have not yet been overcome by non-viral delivery systems.

Similarly, the traditional pollen-tube pathway method, due to its successful delivery of exogenous genes in cotton, is often cited as an alternative. Given the short flowering period of mung bean, this method is theoretically applicable. However, empirical attempts have revealed extremely low actual transformation efficiency and high chimerism rates, necessitating profound optimization before this approach can transition from an experimental exploration into a reliable tool [110].

Furthermore, virus-mediated delivery systems offer another pathway, and recent empirical studies in closely related recalcitrant legumes provide critical insights. For instance, a breakthrough in common bean demonstrated that following the failure of traditional *Agrobacterium tumefaciens* delivery, the delivery of *Bean Yellow Dwarf Virus* replicons via *Rhizobium rhizogenes* successfully achieved CRISPR/Cas9 editing [111]. However, this study also highlighted profound biological barriers: the widely applied *Tobacco Rattle Virus* completely failed to establish infection due to strict host range limitations, and successful gene editing did not translate into actual shoot regeneration due to complex developmental redundancies (e.g., *WOX* genes) [111].

Extrapolating from these findings, engineering legume-compatible viral vectors provides a highly relevant, yet deeply challenging, theoretical pathway for mung bean. While conceptually attractive, empirical application in mung bean currently remains in the hypothetical realm. The severe host range limitations, limited cargo capacity, and the uncoupling of successful cellular editing from whole-plant regeneration underscore that these strategies are speculative frontiers rather than established methodologies. Therefore, while these advanced delivery platforms inspire new “regeneration-bypass” paradigms, their future application demands rigorous empirical validation specifically tailored to the unique biology of *Vigna* species.

### 8.5. Bottlenecks in Paradigm Transfer

In summary, paradigm transfer is far from straightforward and is confronted with three fundamental challenges. Technical Bottlenecks: The low efficiency of genetic transformation in mung bean constitutes a primary obstacle for all subsequent transgenic-based functional validation and precision editing. Biological Complexity: The inherent complexity of polyploid genomes-including the regulation of meiosis, subgenome interactions, and epigenetic memory-far exceeds that of diploids. Direct application of knowledge frameworks developed for simpler models often fails, necessitating a novel cognitive framework [112]. Imbalanced Resource Investment: As an “orphan crop”, mung bean research lacks comparable levels of funding, large-team support, and access to shared high-end research platforms that are available for staple crops. This systemic shortfall in resource investment constrains the feasibility of large-scale, systematic, and high-cost frontier research. Therefore, a successful “paradigm transfer” must entail a profound process of “adaptive re-engineering”. It requires researchers to creatively integrate, streamline, or develop research strategies suited to the specific conditions and practical needs of mung bean.

## 9. Perspectives and Recommendations

### 9.1. Directions for Germplasm Innovation

The future of mung bean improvement lies in the strategic transition from random germplasm innovation to trait-targeted genetic design. A primary direction for future research is the establishment of a “polyploid-mutant dual-drive” composite germplasm repository. Unlike traditional breeding collections, this framework advocates for the systematic integration of artificial polyploidy with large-scale mutagenesis—for instance, by inducing chemical or physical mutations directly into autotetraploid backgrounds. Such a strategy aims to harness the inherent “gigantism” and physiological buffering of polyploids while simultaneously introducing specific allelic variations, such as those governing bruchid resistance or synchronous pod maturity, which remain major bottlenecks in *Vigna* production [113].

### 9.2. Directions for Technological Integration

Regarding technological integration, the community must move toward a high-throughput research paradigm that bridges the gap between induced variation and field application. The deployment of TILLING platforms, integrated with Speed Breeding, offers a powerful route to rapidly identify rare functional mutations within high-density libraries. This is particularly relevant for modifying complex traits like pod indehiscence (non-shattering); by targeting orthologs of dehiscence-related genes (e.g., *Pdh1*) through EMS mutagenesis, researchers can adapt mung bean for once-over mechanical harvesting [93,114]. Furthermore, addressing the genomic complexity inherent in polyploid systems will require the application of Genomic Selection models specifically trained on mutant phenotypic diversity, enabling the early-stage prediction of superior lines before extensive field evaluation.

### 9.3. Application Orientation

Application efforts should be closely aligned with the “mechanized-functional” agricultural transition. Beyond structural traits, the exploitation of mutational variation to enhance grain quality—specifically by targeting the reduction of various anti-nutritional factors (ANFs) such as trypsin inhibitors, lectins, and phytates—will be essential for developing nutritionally superior mung bean varieties. Leveraging induced mutations to disrupt the biosynthesis of these ANFs can mitigate gastrointestinal discomfort and enhance mineral bioavailability, thereby increasing the market value of mung bean in the functional food sector [115]. Simultaneously, leveraging the enhanced root system architecture and stomatal characteristics of polyploid germplasm provides a robust strategy for breeding “climate-smart” varieties capable of stable production on marginal and saline-alkali lands.

### 9.4. Recommendations

To realize these goals, a coordinated international effort is required to overcome the “orphan” status of minor legumes. We recommend the formation of an International *Vigna* Mutagenesis Consortium to standardize phenotypic and genotypic data protocols, creating a shared digital repository of mutant resources. Most critically, long-term funding should be directed toward overcoming the genotype-dependent nature of genetic transformation, which currently limits the precision of gene editing in many minor legumes. By bridging the gap between laboratory-based induction and multi-location ecological testing, the scientific community can establish mung bean as a model for industrial upgrading driven by innovative genetic perturbations.

## 10. Conclusions

This study systematically explores an innovative pathway for advancing the genetic improvement of mung bean and other orphan legume crops by integrating polyploidy and mutation breeding strategies. Although mature research paradigms for polyploidy and mutation breeding have been established in model and major legume crops, transferring these advanced paradigms to mung bean presents unique challenges. These include the widespread and severe fertility barriers in polyploid materials, as well as the slow progress in gene mapping and functional validation within mutation breeding due to technical bottlenecks.

To overcome these barriers, it is imperative to advance the paradigm shift from “random screening” to “precision creation”. The core of this shift lies in constructing a “polyploid-mutant” composite germplasm repository and establishing an integrated, closed-loop research framework of “phenomics-genomics-gene editing”. This requires the standardization and adaptive re-engineering of existing technologies, with a focused effort on tackling fundamental technical bottlenecks such as the low efficiency of genetic transformation in mung bean.

Ultimately, research must be tightly aligned with industrial objectives, specifically the development of breakthrough cultivars that are suitable for mechanization, nutritionally enhanced, and highly resilient. By pursuing this integrated strategy, the breeding practices for mung bean and similar orphan legumes can be guided into a new era of precision design.

## Figures and Tables

**Table 1:** Successful Practices of Different Breeding Methods in Various Crops.

Latin Name	Mutagen/Treatment Method	Mutagenesis/Breeding Outcome
*Glycine max*	EMS	Created large-scale mutant libraries [43].
*Glycine max*	Radiation (γ-rays)	Creation of mutants resistant and tolerant to atrazine [44].
*Glycine max*	CRISPR/Cas9 genome editing	Regulatory factors identified for controlling soybean plant architecture [45].
*Oryza sativa*	Radiation (γ/X-rays)	Obtained allelic variation of the semi-dwarfing gene *sd1*, key to the “Green Revolution” [40].
*Oryza sativa*	EMS	Created mutant populations for breeding traits like early maturity, fragrance, glutinousness [41].
*Oryza sativa*	T-DNA insertion	Constructed a genome-wide insertion mutant library for functional genomics [46].
*Oryza sativa*	CRISPR/Cas9 genome editing	Precisely knocked out the fragrance gene (*BADH2*) to breed aromatic rice varieties [47].
*Triticum aestivum*	Sodium azide (NaN_3_)	Developed mutant lines with enhanced rust resistance [48].
*Triticum aestivum*	EMS + TILLING screening	Increase the β-carotene (provitamin A) content in grains [49].
*Zea mays*	γ-ray irradiation	Obtained high-lysine mutant (*opaque2*), significantly improving nutritional value [50].
*Zea mays*	EMS mutagenesis	Isolated to an extremely internally competed leaf mutant *abrl1* [51].
*Medicago sativa*	EMS	Screened for drought-tolerant mutants [52].
*Vigna sinensis*	Colchicine	It reveals that the meristem can still differentiate ion regulation and metabolism, among other guard cell functions, even when cell division is blocked [53].
*Cicer arietinum*	EMS	A variety resistant to imidazolinone herbicides was developed [54].
*Pisum sativum*	Gamma rays + EMS + CRISPR/Cas system	To enhance pea protein quality and functional genomics research [55].

## Data Availability

No new data were created or analyzed in this study. Data sharing is not applicable to this article. The review does not contain any original data and is based entirely on information presented in previously published studies.

## Abbreviations

The following abbreviations are used in this manuscript:

EMS	Ethyl Methanesulfonate
QTL	Quantitative Trait Locus
TILLING	Targeting Induced Local Lesions IN Genomes
CRISPR/Cas9	Clustered Regularly Interspaced Short Palindromic Repeats/CRISPR-associated protein 9
HTP	High-Throughput Phenotyping

## Appendix A

**Table A1:** Summary of previously published review articles related to legume breeding, genomic tools, and the novel contributions of the present study.

Author & Year	Main Focus/Scope	Key Contributions & Discussed Technologies	Limitations/Gaps Addressed by Our Present Study
Mehandi et al., 2019 [116]	Retrospect and prospects of mung bean breeding	Comprehensively reviewed classical pure-line selection, basic physical/chemical mutation breeding, and early QTL mapping for agronomic traits in mung bean.	Did not cover modern multi-omics integration, CRISPR-based targeted mutagenesis, or the specific mechanisms of artificial polyploidization.
Bohra et al., 2020 [117]	Genomics and molecular breeding in Semi-Arid Tropic (SAT) legumes	Highlighted successful examples of marker-assisted selection (MAS), QTL mapping, and genomic-assisted breeding for disease resistance (e.g., Fusarium wilt) and drought tolerance in chickpea, peanut, and pigeonpea.	Primarily focused on major SAT legumes and molecular markers. Lacked a specific focus on minor legumes (e.g., mung bean) and did not emphasize artificial polyploidization strategies.
Baloglu et al., 2022 [118]	Gene editing technologies (CRISPR/Cas) and transformation in legumes	Provided a comprehensive review of *Agrobacterium*-mediated transformation, CRISPR/Cas9 applications, and delivery systems for trait improvement in leguminous crops.	Heavily biased toward major or model legumes (e.g., soybean, *Medicago*, pea). Did not address the integration of gene editing with classical physical/chemical mutagenesis or polyploidy induction.
Raina et al., 2023 [119]	The transition of legume breeding from conventional approaches to modern omics	Summarized the integration of GWAS, transcriptomics, BSA-seq, machine learning algorithms, and gene editing for improving biotic/abiotic stress tolerance across various legumes.	As a broad editorial overview, it lacked an in-depth mechanistic discussion on artificial polyploidy and the specific translational bottlenecks (paradigm shifts) required exclusively for mung bean.
Majidian, 2022 [120]	Legume breeding: transition from classical to molecular techniques	Comprehensively reviewed classical heritability for agronomic traits, marker-assisted selection (MAS), and traditional genetic modification (GM) traits (e.g., herbicide resistance) in major legumes.	Lacks discussion on modern CRISPR/Cas-based genome editing. Completely omits artificial polyploidy induction mechanisms and the specific translational bottlenecks in minor legumes like mung bean.
Afzal et al., 2019 [121]	Legume genomics, transcriptomics, and computational biology	Reviewed the shift from classical breeding to NGS, emphasizing RNA-sequencing pipelines, metabolomics, and bioinformatics software for gene discovery.	Focused heavily on transcriptomic pipelines for general legumes. Lacked specific discussions on combining these omics tools with artificial ploidy manipulation.
The Present Study	Polyploidy & Mutagenic Innovation in Minor Legumes (Mung Bean)	Integrates classical polyploid induction and physical/chemical mutagenesis with modern multi-omics and gene-editing paradigms.	Explicitly bridges the translational gap from model crops to mung bean. Establishes a synergistic framework combining ploidy manipulation with advanced genomic tools.
